# Supplementary material for: Chronoradiobiology of Breast Cancer: The Time Is Now to Link Circadian Rhythm and Radiation Biology
Source: Int J Mol Sci. 2022 Jan 25;23(3):1331. doi: 10.3390/ijms23031331 (PMC8836288; doi:10.3390/ijms23031331)
Supplement: Supplementary file 1 [file ijms-23-01331-s001.zip › ijms-1561540-supplementary.pdf]

## Supplementary Materials

**Table S1.** Effect of time of day on radiation treatments for non-breast cancers. Any overall conclusions about a preferred radiation time are indicated in bold in the findings. See Table 3 for studies on the effect of time of day on radiation treatments for breast cancer. SRS = stereotactic radiosurgery, PBT = proton beam therapy.

| Time of Radiotherapy for Other Cancers | | | | |
|---|---|---|---|---|
| **Cancer Site and Study** | **Type** | **Timing** | **Radiation** | **Endpoints and Findings** |
| Head and neck<br>Oral mucositis in morning vs. evening irradiated patients: a randomized prospective study | Prospective randomized (n = 212) | 8:00–11:00 vs. 15:00–18:00 | Daily 2-Gy fractions 5×/week > 60 Gy with Cobalt- 60 | Oral mucositis:<br>Trend toward increased grade 3 toxicity of mucositis in the evening group (not significant, p =0.08)<br>(Early preference) |
| Head and neck<br>Comparison of toxicity associated with early morning versus late afternoon radiotherapy in patients with head-and-neck cancer: a prospective randomized trial of the National Cancer Institute of Canada Clinical Trials Group (HN3) | Prospective randomized (n = 205) | 8:00–10:00 vs. 16:00–18:00 | 66–70 Gy in 3–35 fractions | Grade 3 mucositis:<br>Grade 3 mucositis higher in evening group (62% vs. 53%) for all patients (not significant, p = 0.17)<br>Grade 3 mucositis significantly higher in evening group for smokers receiving >66 Gy (p = 0.024)<br>Less weight loss noted in the morning group<br>(Early preference) |
| Head and neck<br>The impact of delivery daytime and seasonality of radiotherapy for head and neck cancer on toxicity burden | Retrospective (n = 617) | Seasonality:<br>Median day of radiotherapy falling after March equinox (“light”) vs. after September equinox (“dark”)<br>Time of day: median radiotherapy time before vs. after 12:00 | ≥60 Gy, definitive or adjuvant, ±chemotherapy | Acute toxicity, late toxicity:<br>Higher acute toxicity with radiotherapy delivered in the “dark” half of the year (p = 0.0127)<br>Time of day was not associated with toxicities analyzed; trend toward higher acute toxicity in the afternoon (p = 0.0387, did not meet authors’ criteria)<br>(Early preference) |
| Cervical<br>Circadian variation in radiation-induced intestinal mucositis in patients with cervical carcinoma | Prospective randomized (n = 229) | 8:00–10:00 vs. 18:00–20:00 | 50 Gy in 25 fractions using Cobalt- 60 | Multiple toxicities:<br>Increased grade 3+ diarrhea in the morning group (p < 0.01)<br>Other toxicities increased in morning group (not significant)<br>(Late preference) |
| Cervical<br>Research on radiotherapy at different times of the day for inoperable cervical cancer | Prospective randomized (n = 67) | 9:00–11:00 vs. 21:00–23:00 | 50 Gy in 25 fractions external beam and 36–42 Gy in 6–7 fractions brachytherapy | GI toxicity, hematological toxicity, apoptotic potential:<br>Increased grade 3+ GI toxicity in the morning group (75% vs. 58% diarrhea, 13% vs. 6% high-grade toxicity)<br>Worse hematologic toxicity in afternoon group<br>Levels of PER1, PER2, CLOCK correlated with apoptosis<br>(Ambivalent preference) |

| | | | | |
|---|---|---|---|---|
| Prostate<br>Differences in toxicity and outcome associated with circadian variations between patients undergoing daytime and evening radiotherapy for prostate adenocarcinoma | Retrospective (n = 409) | Before vs. after 17:00 | High-dose radiotherapy (median dose 78 Gy without elective pelvis treatment) | Toxicity, failure-free survival:<br>Increased grade 2+ late GI toxicity for patients older than 70 in the afternoon group ($p < 0.001$)<br>Poorer 5-year failure-free survival for afternoon group ($p = 0.05$)<br>(Early preference) |
| Prostate<br>Chronoradiation Therapy for Prostate Cancer: Morning Proton Beam Therapy Ameliorates Worsening Lower Urinary Tract Symptoms | Prospective cohort (n = 168) | 08:30–10:30 vs. 10:31–14:30 vs. 14:31–16:30 | PBT 78 Gy in 39 fractions or 70 Gy in 28 fractions | Urinary tract symptoms, quality of life:<br>Morning PBT ameliorated quality of life and lower urinary tract symptoms from localized prostate cancer compared to near-noon or late afternoon groups under multiple linear regression ($p < 0.05$)<br>(Early preference) |
| Rectal<br>Does chronomodulated radiotherapy improve pathological response in locally advanced rectal cancer? | Retrospective (n = 155) | Before vs. after 12:00 | 25–50.4 Gy neo-adjuvantly | Tumor response, staging:<br>Increased complete pathologic response ($p = 0.035$) and nodal downstaging in afternoon group<br>(Late preference) |
| Brain (high-grade glioma)<br>Clinical effects of morning and afternoon radiotherapy on high-grade gliomas | Retrospective (n = 109) | Before vs. after 12:00 | 60 Gy conventional fractionation, hypo fractionation for frail patients | Local control, distant CNS control, progression-free survival, overall survival:<br>No difference in overall survival ($p = 0.76$) or progression free survival ($p = 0.30$)<br>Univariate analysis showed improved overall survival if treated in the morning ($p = 0.05$)<br>(Early preference) |
| Brain metastases<br>Could time of whole brain radiotherapy delivery impact overall survival in patients with multiple brain metastases? | Retrospective (n = 755) | 8:00–11:00 vs. 11:00–14:00 vs. 14:00–17:00 | WBRT, 30 Gy in 10 fractions (93%) and 20 Gy in 5 fractions | Overall survival:<br>No difference in overall survival for total group<br>Univariate analysis showed survival difference for elderly women with ≥70% or ≥80% WBRT received in a single time window ($p = 0.02$)<br>(Preference for consistent timing) |
| Brain metastases from lung (NSCLC)<br>Impact of time of day on outcomes after stereotactic radiosurgery for non-small cell lung cancer brain metastases | Retrospective (n = 437) | 9:12–11:40 vs. 11:41–18:02 (determined with receiver operating characteristics analysis) | SRS 18–20 Gy | Overall survival, local control:<br>Univariate analysis showed decreased overall survival ($p = 0.016$) and local control ($p = 0.012$) in the later time group<br>Effect not reproduced on multivariate analysis with confounders<br>(Early preference) |
| Brain metastases from lung (NSCLC)<br>The effect of treatment time on outcome in non-small cell lung | Retrospective (n = 172) | Before vs. after 12:00 | SRS treatment to 1 lesion, median dose 21 Gy | Overall survival, local control:<br>No difference in overall survival or local control seen; linac-based SRS |

| | | | | |
|---|---|---|---|---|
| cancer brain metastases treated with stereotactic radiosurgery | | | | can be effectively delivered in the morning or afternoon (No preference) |
| Brain metastases from lung (NSCLC) Gamma knife radiosurgery for brain metastasis of non-small cell lung cancer: is there a difference in outcome between morning and afternoon treatment? | Retrospective (n = 97) | 10:00–12:30 vs. 12:30–15:00 | SRS with GammaKnife, mean dose 18.6 Gy | Survival, CNS vs. systemic cause of death: Improved local control and overall survival for morning group (p = 0.014, 0.025) (Early preference) |
| Bone metastases Effects of circadian rhythms and treatment times on the response of radiotherapy for painful bone metastases | Retrospective (n = 194) | 8:00–11:00 vs. 11:00–14:00 vs. 14:00–17:00 | 8 Gy, 20 Gy, 30 Gy | Pain palliation: Improved pain control (clinical response) if treated 11:00 to 14:00 group, detected for females only (p = 0.02) (Midday preference) |
| Nasopharyngeal, Esophageal, Lung, Cervical The clinical effect study on malignant tumors with chronoradiotherapy | Prospective randomized (n = 121) | 9:00 vs. 21:00 | Radiotherapy regimens varied by disease | Toxicity, short-term tumor shrinkage: Evening radiation gave significantly lower toxicity for all cancers Evening radiation significantly increased tumor shrinkage for esophageal, cervical; similar nonsignificant trend for lung, nasopharyngeal (Late preference) |

**Table S2.** Zeitgeber-driven chronoradiotherapy. Hypothetical individualized regimen that synergizes light, clock-acting medication, and diet to improve breast cancer response to radiation treatment and patient outcome.

| **Hypothetical Cancer with Optimal Radiation Therapeutic Index at 12:00h; Patient's Fractionated Radiation Treatments Scheduled for 09:00h Daily** | | |
|---|---|---|
| **Light–Dark** | **Chronopharmaceutical** | **Dietary** |
| 30 min blue light before bedtime (until DLMO shifts to 03:00h) | 20 mg tasimelteon every morning (until DLMO shifts to 3:00h) | Timed feeding such that midday falls at 15:00h |
| Avoid bright light in the morning | Fix 24-h period with KL001 | Intermittent fasting between meals |
| Confirm patient's circadian phase with biomarkers (e.g., clock gene expression) at 09:00h each session just before radiation | | |
